# Therapeutic potential of simvastatin in ALS: Enhanced axonal integrity and motor neuron survival through Apoa4 and Alb modulation

**DOI:** 10.17305/bb.2024.11218

**Published:** 2024-11-20

**Authors:** Song Luo, Xiaorui Wang, Bo Ma, Dongliang Liu, Li Li, Lijin Wang, Ning Ding, Liangyu Zou, Jie Wang, Jialin Pan, Daoqian Sang, Huadong Zhou, Hongdang Qu, Yi Lu, Lijuan Yang

**Affiliations:** 1 Department of Neurology, The First Affiliated Hospital of Bengbu Medical University, Bengbu, China; 2 Department of Psychiatry, Bengbu Medical University, Bengbu, China; 3 Department of Hematology, The First Affiliated Hospital of Bengbu Medical University, Bengbu, China; 4 Department of Neurology, Shenzhen People’s Hospital, The Second Clinical Medical College, Jinan University, Shenzhen, China; 5 International Medical Center (Department of Geriatric Medicine), Shenzhen University General Hospital, Shenzhen, China; 6 Department of Internal Medicine, Second People’s Hospital, Longgang District, Shenzhen, China; 7 Department of Pediatrics, The First Affiliated Hospital of Bengbu Medical University, Bengbu, China

**Keywords:** Simvastatin, amyotrophic lateral sclerosis, transgenic mouse model, Apoa4, Alb

## Abstract

Amyotrophic lateral sclerosis (ALS) is a neurodegenerative disease characterized by the selective death of motor neurons in the spinal cord, brainstem, and motor cortex. This study investigates the effects of simvastatin on the G93A-copper/zinc superoxide dismutase (G93ASOD1) transgenic mouse model of ALS. The experiment included three groups: C57BL/6 wild-type mice, C57BL/6J SOD1^G93A^ mice treated with PBS (SOD1^G93A^ + PBS), and C57BL/6J SOD1^G93A^ mice treated with simvastatin (SOD1^G93A^ + simvastatin). The primary endpoints were survival rates, body weight changes, performance in pole climbing and suspension tests, and neurological deficit scores. Pathological changes were assessed using hematoxylin and eosin staining, transmission electron microscopy, Nissl staining, and Masson staining. Proteomic and metabolomic analyses were performed to identify differentially expressed proteins (DEPs) and metabolites. Quantitative real-time polymerase chain reaction and western blotting were used to measure gene expression. Although there were no significant differences in survival rates, body weight, pole climbing, and suspension test performance, or neurological deficit scores between the SOD1^G93A^ + simvastatin and SOD1^G93A^ + PBS groups, simvastatin treatment improved axonal organization within the spinal cord, increased the number of neurons, and reduced cytoplasmic swelling and gastrocnemius fibrosis. A total of 47 DEPs and 13 differential metabolites were identified between the SOD1^G93A^ + PBS and SOD1^G93A^ + simvastatin groups. Notably, the expression levels of Apoa4 and Alb were elevated in the SOD1^G93A^ + simvastatin group compared to the SOD1^G93A^ + PBS group. Our results suggest that simvastatin may have potential therapeutic effects in ALS, likely involving the modulation of Apoa4 and Alb expression.

## Introduction

Amyotrophic lateral sclerosis (ALS), also known as Lou Gehrig’s disease, is an incurable and devastating neurodegenerative condition characterized by the degeneration of motor neurons in the upper and lower limbs [1]. This degeneration leads to somatic muscle dysfunction and ultimately results in respiratory failure. The most common symptoms of ALS are fatigue and reduced motor function. Additionally, a subset of ALS patients exhibits frontotemporal lobe degeneration (FTLD/FTD), the second most common form of dementia after Alzheimer’s disease [2]. Approximately 10% of ALS cases are classified as familial, while the remaining 90% are sporadic [3, 4]. The genetic mechanisms underlying ALS development are typically characterized by complex interactions between multiple genes and molecules [5]. Although there is currently no cure for ALS, two drugs have been approved for treatment [6].

Simvastatin is a long-established hydroxy-methylglutaryl coenzyme A (HMG-CoA) reductase inhibitor [7]. It is widely used in the prevention and treatment of cardiovascular and cerebrovascular diseases due to its cholesterol-lowering and anti-inflammatory effects [8]. Recent studies suggest that simvastatin may also be effective in treating tumors, such as breast cancer, ovarian cancer, and colon cancer [9–11]. However, the use of simvastatin in the treatment of ALS and other neuromuscular diseases remains controversial. The Apoa4 gene encodes apolipoprotein A-IV, a protein that undergoes hydrolysis and glycosylation to form acidic glycoproteins [12]. The Apoa4 gene plays a significant role in lipid transport and metabolism, particularly in cholesterol reversal [13, 14]. Extensive research conducted both domestically and internationally has demonstrated a correlation between Apoa4 and levels of plasma lipoprotein, cholesterol, and triglycerides [13, 15–17]. Alb is an essential plasma protein produced by hepatocytes [18]. A previous study indicated that Alb levels decreased in 25% of ALS patients, while in most ALS patients, Alb levels either increased or remained unchanged [19].

The superoxide dismutase 1 (SOD1) gene was the first mutated gene identified in ALS. Mice with mutations in the human SOD1 gene exhibit clinical manifestations similar to those of ALS patients, with pathological evidence of comparable neuronal loss. As a classic ALS animal model, SOD1^G93A^ transgenic mice are widely used in pathogenesis research and drug development [20].

In this study, we utilized an ALS model using C57BL/6J SOD1G93A mice. The effect of simvastatin on ALS was analyzed in depth through proteomics and metabolomics, aiming to enhance the scientific foundation for the clinical application of simvastatin in ALS treatment.

## Materials and methods

### Mice model

**Figure S1.**
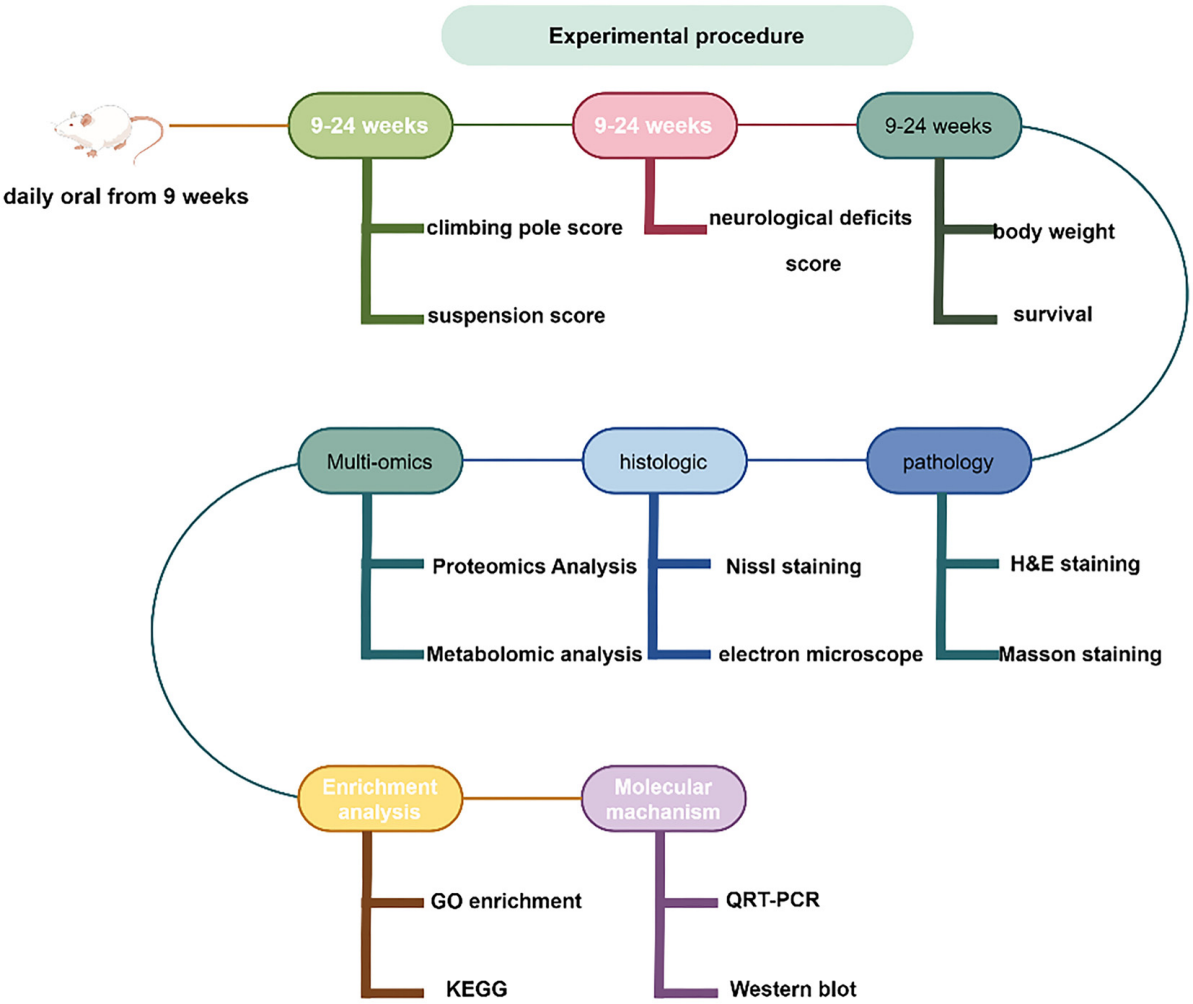
**Experimental procedure for this study.** H&E: Hematoxylin and eosin; qRT-PCR: Quantitative real-time polymerase chain reaction; GO: Gene Ontology; KEGG: Kyoto Encyclopedia of Genes and Genomes.

The animals used in this study were C57BL/6J SOD1^G93A^ transgenic mice and littermate C57BL/6 wild-type (WT) mice, aged 4–6 weeks, purchased from Jackson Laboratory. After one week of adaptive feeding, the C57BL/6J SOD1^G93A^ mice were randomly assigned to two groups: the SOD1^G93A^ + PBS group and the SOD1^G93A^ + simvastatin group (*n* ═ 6). The SOD1^G93A^ + simvastatin group received a daily oral dose of 20 mg/kg simvastatin (MSD, Zhejiang, China), dissolved in 5% carboxymethyl cellulose sodium (CMC-Na), starting from the ninth week via gavage [4]. The SOD1^G93A^ + PBS group received a daily oral dose of 0.2 mL of PBS. The mice were housed in a pathogen-free environment with unrestricted access to food and water. SOD1^G93A^ mice that were unable to maintain an upright position within 30 s of being placed in a supine position were subjected to endpoint treatment. Beginning in the ninth week, the body weights of the mice were measured weekly, transitioning to thrice-weekly measurements starting from the 20th week. All animals were used for behavioral testing, and for other experiments, at least three animals per group were included. The experimental procedure is illustrated in Figure S1.

### Behavioral test

The mice were subjected to a weekly measurement from the ninth week, which was subsequently increased to twice a week from week 12. Each mouse was tested three times per session, with each experiment lasting 5 min and a 10-min interval between tests. An inability to sustain the maximum running time of 5 min during motor function testing was recorded as disease onset. In the pole climbing test, reaching the midpoint of the wooden pole within 3 s was scored as three points, achieving this within 6 s was scored as two points, and exceeding 6 s was scored as one point. The mice underwent three trials, and the mean value was calculated for each mouse. In the hanging test, the front paws of the mouse were suspended horizontally on a line. A score of three points was awarded if both paws were able to hang, two points if only one paw could hang, and one point if neither paw could sustain the suspension. Each test was performed three times.

### Neurological deficit scores

Neurological deficits were assessed using a scoring system: four points indicated normalcy (absence of motor dysfunction), three points indicated significant hind limb tremors upon tail suspension, two points indicated gait abnormalities, one point indicated walking with dragging of at least one hind limb, and zero points indicated an inability to stand independently within 30 s.

### Hematoxylin and eosin (H&E) staining

The brainstem tissue was fixed for 24 h, embedded in paraffin wax, and sectioned into slices 4 µm thick. After dewaxing with water, the sections underwent H&E staining (Servicebio, Wuhan, China), followed by dehydration and sealing. The stained sections were observed under a microscope (Olympus, Japan), and images were collected for analysis.

### Nissl staining

The tissue slices were dewaxed and washed with distilled water, then immersed in tar purple staining solution and maintained at 56 ^∘^C for staining. After rinsing thoroughly with distilled water, the slices were treated with Nissl differentiation solution (ebiogo, China) for a few seconds and observed under a microscope until the background appeared nearly colorless. The slices were then subjected to routine dehydration, treated with xylene for transparency, sealed with neutral gum (ebiogo, China), and observed microscopically. Neurons were identified and counted based on Nissl morphology using ImageJ software.

### Masson staining

Sections were routinely dewaxed in a gradient of alcohol solutions down to water. Bouin solution was added, and the sections were incubated in a 37 ^∘^C incubator, then rinsed with running water until the yellow color disappeared. Azure blue staining solution (ebiogo, China) was applied for 3 min, followed by washing with water. Mayer’s hematoxylin staining solution was applied for 3 min, after which the sections were washed with water. Differentiation was performed with acidic ethanol for a few seconds, followed by thorough rinsing with running water. Magenta staining solution was applied for 10 min and rinsed slightly with distilled water. The sections were treated with phosphomolybdic acid solution for 5 min, after which the supernatant was removed, and aniline blue staining solution was added for 5 min. After weak acid treatment, the sections were subjected to conventional dehydration, xylene transparency, and sealed with neutral gum (ebiogo, China). A microscope (CX41, Olympus, Japan) was used to observe changes in the gastrocnemius muscle. The collagen volume fraction was calculated using ImageJ software.

### Transmission electron microscope

ySpinal cord tissue was fixed using an electron microscope-grade fixative. Following dehydration at ambient temperature, the specimen was infiltrated, embedded, and polymerized at 60 ^∘^C for 48 h. The resulting resin block was sectioned into ultrathin slices (60–80 nm) using an ultramicrotome. The ultrathin slices were placed onto 150-mesh square membrane copper grids, stained, and examined under a transmission electron microscope. Images were collected for further analysis.

### Proteomics analysis

The peptides were labeled using tandem mass tag (TMT) reagents following the manufacturer’s instructions (Thermo Fisher Scientific). Each aliquot containing 100 µg of peptide equivalent was reacted with one tube of TMT reagent. The sample was dissolved in 100 µL of triethylammonium bicarbonate (TEAB) buffer (pH 8.5), and the TMT reagent was dissolved in 41 µL of water-free acetonitrile. The mixture was left at ambient temperature for 1 h. Subsequently, 8 µL of 5% hydroxylamine was added to the sample and incubated for 15 min to stop the reaction. The multiplex-labeled samples were combined and subjected to lyophilization.

The TMT-labeled peptide mixture was fractionated using a Waters XBridge BEH130 column (C18, 3.5 µm, 2.1 × 150 mm) on an Agilent 1290 HPLC operating at a flow rate of 0.3 mL/min. Buffer A consisted of 10 mM ammonium formate, while buffer B consisted of 10 mM ammonium formate mixed with 90% acetonitrile. Both buffers were adjusted to pH 10 using ammonium hydroxide. For each peptide mixture, 30 fractions were collected and subsequently pooled into 15 fractions by combining equal-interval RPLC fractions.

LC-MS was performed on a Q Exactive mass spectrometer (Thermo Fisher Scientific, USA). Peptides from each fraction were introduced onto a C18 reversed-phase column (12 cm length, 75 µm inner diameter, 3 µm particle size) using buffer A, consisting of 2% acetonitrile and 0.1% formic acid. Separation was achieved with a linear gradient of buffer B, composed of 90% acetonitrile and 0.1% formic acid, at a flow rate of 300 nL/min for 90 min. The gradient was adjusted as follows:

0–2 min: 2%–5% buffer B, 2–62 min: 5%–20% buffer B, 62–80 min: 20%–35% buffer B, 80–83 min: 35%–90% buffer B, and 83–90 min: 90% buffer B maintained MS data acquisition used a data-dependent top-15 approach, dynamically selecting the most prevalent precursor ions from the survey scan (300–1800 m/z) for high-energy collision dissociation (HCD) fragmentation. Predictive automatic gain control (pAGC) was employed to determine target values. For full MS, the AGC target value was set to 1e6 with a maximum injection time of 50 ms, while for MS2, the AGC target value was set to 1e5 with a maximum injection time of 100 ms. Dynamic exclusion duration was set to 30 s. Survey scans were acquired at a resolution of 70,000 at m/z 200, while HCD spectra were recorded at a resolution of 35,000 at m/z 200. The collision energy was set to 30 units. Peptide recognition mode was enabled during instrument operation.

### Differentially expressed proteins (DEPs) database analysis

LC-MS/MS raw files were processed in Proteome Discoverer 2.4 software (version 1.6.0.16) for data interpretation and protein identification from the database. Bioinformatics data analysis was performed using Perseus software, Microsoft Excel, and R statistical computing software. DEPs were identified based on a fold-change (FC) ratio greater than 1.2 or less than 1/1.2 and a *P* value < 0.05. Hierarchical clustering grouped expression data based on protein levels.

Protein sequence annotation was performed using data from UniProtKB/Swiss-Prot, Gene Ontology (GO), and the Kyoto Encyclopedia of Genes and Genomes (KEGG). GO and KEGG enrichment analyses were conducted using Fisher’s exact test, with false discovery rate (FDR) correction for multiple testing. GO terms were categorized into biological process (BP), molecular function (MF), and cellular component (CC). Enriched GO and KEGG pathways were considered statistically significant at *P* < 0.05. The STRING database and Cytoscape software were used to construct protein–protein interaction (PPI) networks.

### Metabolomic analysis

Metabolomic analysis was conducted by Metabo-Profile Biotechnology (Shanghai, China) according to previous studies [21, 22]. An ACQUITY UPLC-Xevo TQ-S system (Waters Corp., USA), an ultra-performance liquid chromatography coupled to tandem mass spectrometry system (UPLC-MS/MS, Waters Corp., Milford, MA, USA), was used to measure metabolites in the tissue samples. Metabolites were identified by comparison with an internal library built using standard reference chemicals.

Metabolomic analysis involved examining potential bioinformatics data, applying a significance level of *P* < 0.05 and a minimum log2FC of 0. Retention times (RTs) were linearly shifted throughout the run using internal standard normalization methods. Peak annotation processing was performed using an in-house MS/MS database. Orthogonal projections to latent structure discriminant analysis (OPLS-DA) models were used to visualize differences among the three groups in both positive and negative ion modes. The importance (VIP) values of the variables in the projection were obtained from each variable in the OPLS-DA model. Metabolites were analyzed using the nonparametric Mann–Whitney *U* test, with correction for multiple testing performed using the Benjamini–Hochberg method. Metabolites with VIP values > 1.0 and *P* < 0.05 were considered statistically significant and were collected from the Small Molecule Pathway Database (SMPDB). Pathway enrichment analysis of differential metabolites was conducted using Metabolite Set Enrichment Analysis (MSEA).

### Quantitative real-time polymerase chain reaction (qRT-PCR)

Total RNA from spinal cord tissues was isolated using the AG RNAex Pro RNA kit (Agbio, Changsha, China). Complementary DNA (cDNA) was synthesized using the Evo M-MLV kit (Agbio, Changsha, China). qRT-PCR was performed using SYBR Green Pro Taq HS (Agbio, Changsha, China) on a StepOnePlus™ system (ABI, USA). Primer sequences are listed in Table S1. Reaction conditions were as follows: 95 ^∘^C for 30 s, followed by 40 cycles of 95 ^∘^C for 5 s and 60 ^∘^C for 30 s. The mRNA expression levels were calculated using the 2^-ΔΔCt^ method.

### Western blot

Total protein from spinal cord tissues was extracted using radioimmunoprecipitation assay (RIPA) lysis buffer (Solarbio, Beijing, China) supplemented with phenylmethanesulfonyl fluoride. Protein concentration was determined using a bicinchoninic acid (BCA) kit (Beyotime, Shanghai, China). Protein lysates were separated on 8%–12% SDS-PAGE (Epizyme Biotech, Shanghai, China). A 40 µg sample was loaded into each well for analysis. Proteins were transferred to polyvinylidene fluoride (PVDF) membranes (Millipore, USA) and blocked with 5% nonfat dry milk solution. The membranes were incubated with primary antibodies (detailed below), followed by goat anti-rabbit IgG horseradish peroxidase (HRP) secondary antibody (1:10,000, abs20040, Absin, Shanghai, China). Signals were visualized using an enhanced chemiluminescence (ECL) kit (Proteintech, Wuhan, China). Results were normalized to the loading control GAPDH and quantified using Fusion software.

Primary antibodies: C4a (1:3000, 22233-1-AP, Proteintech, Wuhan, China); C5 (1:600, A8104, Abclonal, Wuhan, China); C3 (1:10,000, 21337-1-AP, Proteintech); PI3K (1:1000, ab302958, Abcam, UK); p-PI3K p85 alpha (Tyr607) (1:1000, AF3241, Affinity, Jiangsu, China); Alb (1:10,000, 16475-1-AP, Proteintech); p-AKT (Ser473) (1:10,000, 66444-1-Ig, Proteintech); AKT (1:5000, 60203-2-Ig, Proteintech); PPARγ (1:5000, 16643-1-AP, Proteintech); APOA4 (1:8000, 17996-1-AP, Proteintech); C1qB (1:1000, A5339, Abclonal, Wuhan, China); CRP (1:2000, 66250-1-Ig, Proteintech); and GAPDH (1:10,000, 60004-1-Ig, Proteintech).

### Ethical statement

All animal experiments were conducted in compliance with the guidelines for the Care and Use of Laboratory Animals from the National Institutes of Health (NIH Pub. No. 85-23; revised 1996). Approval for the study was obtained from the Ethics Committee of the First Affiliated Hospital of Bengbu Medical University (Approval number: 2022282).

### Statistical analysis

GraphPad Prism v9.0 was used for data analysis and visualization. Data are presented as mean ± standard deviation (SD). One-way analysis of variance (ANOVA) was conducted to assess overall mean differences among groups, followed by the least significant difference (LSD) test for pairwise comparisons. *P* < 0.05 was considered statistically significant.

## Results

### Neurological scores and motor function scores of mice after simvastatin treatment

**Figure 1.**
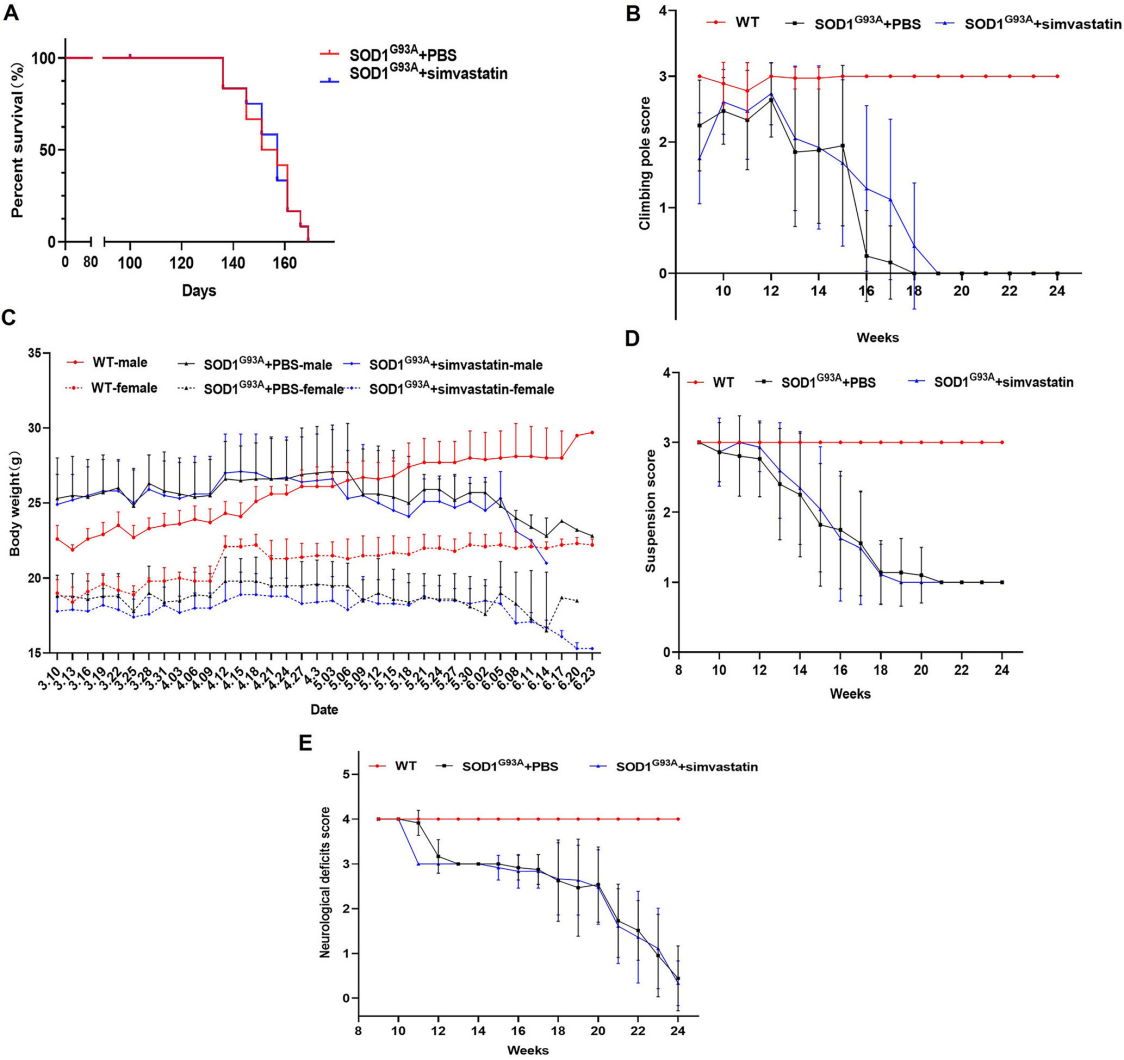
**The effect of simvastatin on phenotype, motor, and neurological deficit scores in mice.** (A) The survival of mice in different groups showed no difference. Climbing pole scores (B), body weight (C), suspension score (D), and neurological deficits score (E) in the SOD1^G93A^ + PBS and SOD1^G93A^ + simvastatin groups were decreased compared to the WT group. SOD1: Superoxide dismutase 1; WT: Wilt-type.

From day 140 to day 160, the survival rate of the SOD1^G93A^ + simvastatin group was higher than that of the SOD1^G93A^ + PBS group (Figure 1A). There was no significant difference in the pole climbing test scores between the SOD1^G93A^ + simvastatin group and the SOD1^G93A^ + PBS group from weeks 10 to 14. The decline in scores in the SOD1^G93A^ + simvastatin group was slower than in the SOD1^G93A^ + PBS group from weeks 16 to 18 (Figure 1B). No significant differences in body weight, suspension test results, or neurological deficit scores were observed between the SOD1^G93A^ + simvastatin group and the SOD1^G93A^ + PBS group (Figure 1C–1E).

### Pathological changes in spinal cord tissues and gastrocnemius

**Figure 2.**
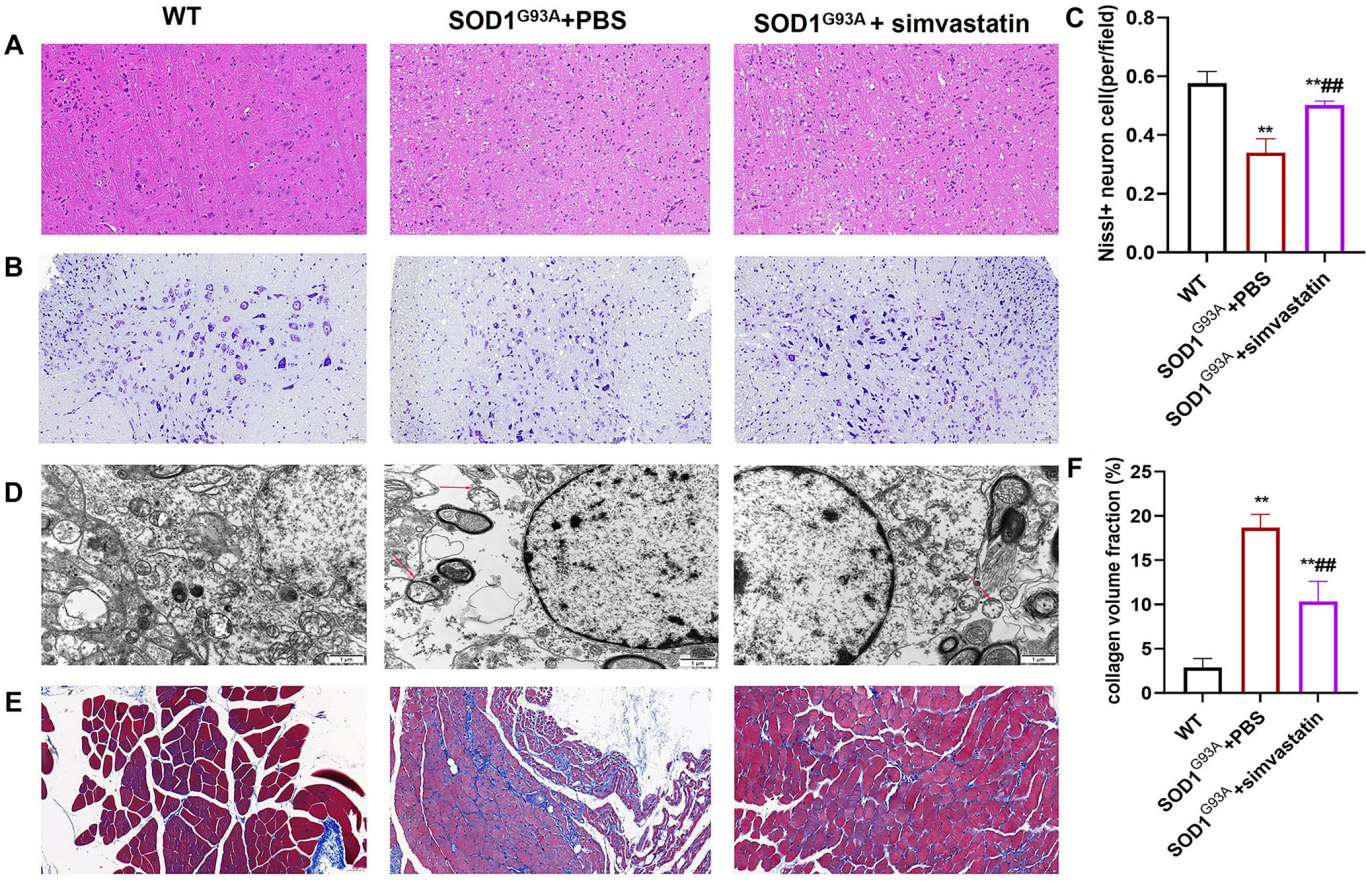
**Simvastatin improves pathological changes in spinal cord tissue and gastrocnemius muscle.** (A) Pathological changes in the brainstem were more severe in the SOD1^G93A^ + PBS group compared to the WT and SOD1^G93A^ + simvastatin groups; (B) Spinal neurons in the SOD1^G93A^ + PBS group showed a reduction in Nissl bodies; (C) Bar graph showing changes in neuron numbers determined by Nissl staining; (D) Vacuolar degeneration of mitochondrial cristae (red arrow) was evident in the SOD1^G93A^ + PBS group; (E) Fibrosis in gastrocnemius muscle tissue was observed in the SOD1^G93A^ + PBS group; (F) Collagen volume fraction was calculated using Image J software. **P < 0.01 vs WT group; ^##^*P* < 0.01 vs SOD1^G93A^ + PBS group. SOD1: Superoxide dismutase 1; WT: Wilt-type.

The SOD1^G93A^ + PBS group exhibited a significantly increased number of intercellular vacuoles in the brainstem, disrupted axons, a significant loss of large anterior horn cells, and increased interstitial swelling in the spinal cord compared to the WT group. After simvastatin treatment, axons became relatively orderly, the number of anterior horn motor neurons increased, and cytoplasmic swelling was reduced. Additionally, Nissl staining revealed that the SOD1^G93A^ + simvastatin group had more neurons than the PBS group (Figure 2A–2D). Masson staining showed that the degree of gastrocnemius fibrosis was significantly increased in the SOD1^G93A^ + PBS group compared to the WT group, whereas gastrocnemius fibrosis was reduced in the SOD1^G93A^ + simvastatin group compared to the SOD1^G93A^ + PBS group (Figure 2E and 2F, *P* < 0.01).

### DEPs in spinal cord tissues of mice from different groups

**Figure 3.**
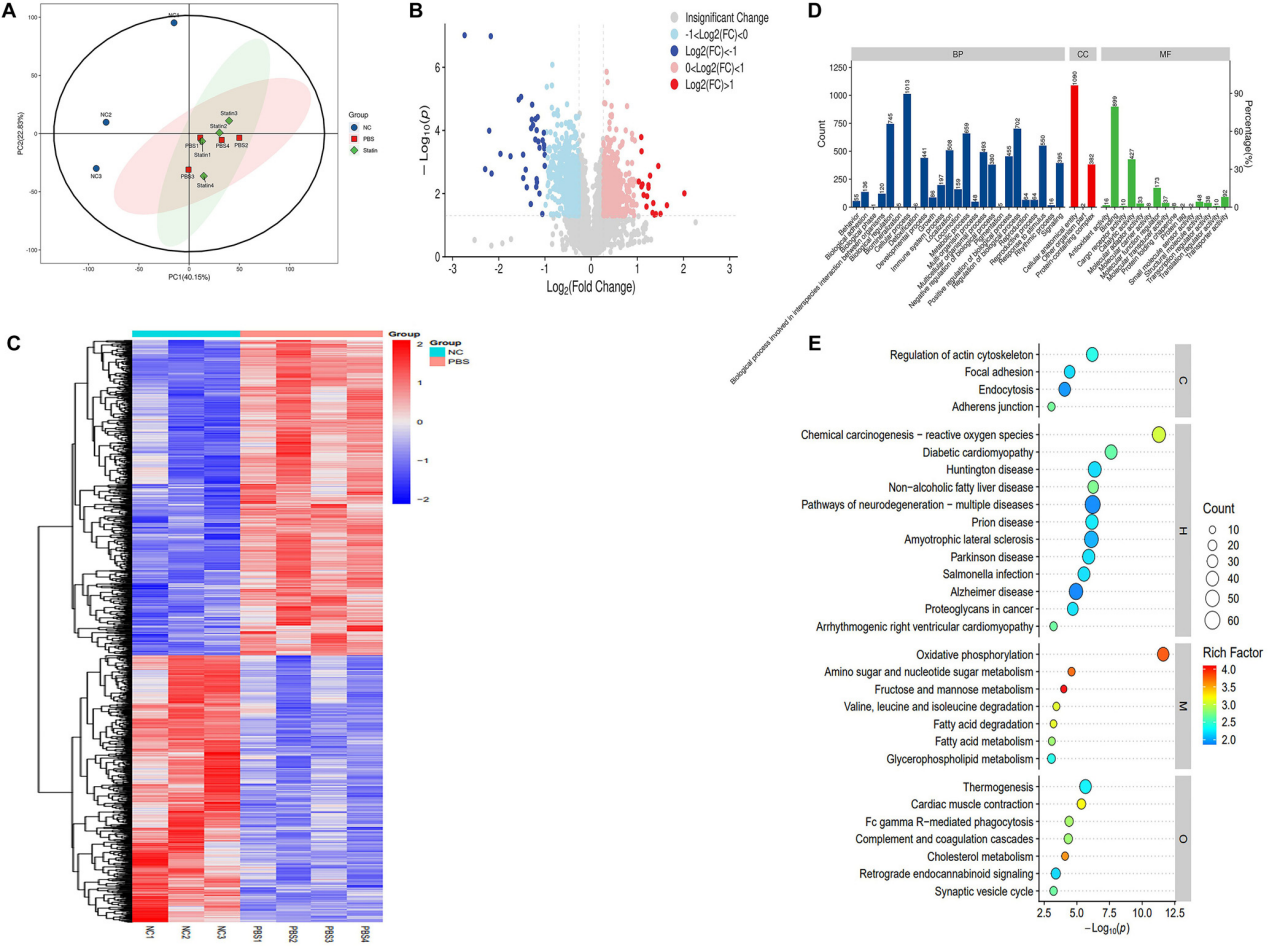
**Proteomic profile shift in spinal cord tissues of mice between the WT and SOD1^G93A^ + PBS groups.** (A) Principal component analysis showing structural results among three groups; (B) Volcano plot displaying altered proteins. Red dots indicate significantly upregulated proteins, while blue dots indicate significantly downregulated proteins in the WT group compared to the SOD1^G93A^ + PBS group; (C) Heatmap showing differentially expressed proteins between the WT and SOD1^G93A^ + PBS groups. The red band represents upregulated protein levels, while the blue band represents downregulated protein levels in the WT group compared to the SOD1^G93A^ + PBS group; (D) GO function annotation diagram for DEPs; (E) KEGG pathway enrichment results show that DEPs were primarily enriched in oxidative phosphorylation, amino sugar, and nucleotide sugar metabolism. SOD1: Superoxide dismutase 1; WT: Wilt-type; FC: Fold-change; GO: Gene Ontology; DEPs: Differentially expressed proteins; KEGG: Kyoto Encyclopedia of Genes and Genomes.

Principal component analysis (PCA) revealed distinct differences among the WT (NC) group, SOD1^G93A^ + PBS group, and SOD1^G93A^ + simvastatin group (Figure 3A). A total of 1130 DEPs were identified, including 519 upregulated and 611 downregulated proteins in the SOD1^G93A^ + PBS group compared to the NC group. Proteins with notable regulation between the SOD1^G93A^ + PBS and WT groups are depicted in Figure 3B. Clustering analysis revealed distinct patterns significantly enriched between the SOD1^G93A^ + PBS and WT groups (Figure 3C).

GO function annotation demonstrated that these DEPs primarily participated in BPs, including cellular processes, biological regulation, regulation of BPs, metabolic processes, response to stimulus, localization, multicellular organism processes, positive regulation of biological regulation, developmental processes, signaling, and negative regulation of biological regulation. CC annotations included cellular anatomical entities and protein-containing complexes, while MF annotations included binding, catalytic activity, and MF regulation (Figure 3D).

KEGG pathway analysis revealed significant involvement (*P* < 0.05) in pathways, such as regulation of actin cytoskeleton, focal adhesion, chemical carcinogenesis-reactive oxygen species, diabetic cardiomyopathy, Huntington’s disease, non-alcoholic fatty liver disease, prion disease, Parkinson’s disease, oxidative phosphorylation, amino sugar and nucleotide sugar metabolism, fructose and mannose metabolism, valine, leucine, and isoleucine degradation, fatty acid degradation, fatty acid metabolism, cardiac muscle contraction, Fc gamma R-mediated phagocytosis, complement and coagulation cascades, and others (Figure 3E).

**Figure 4.**
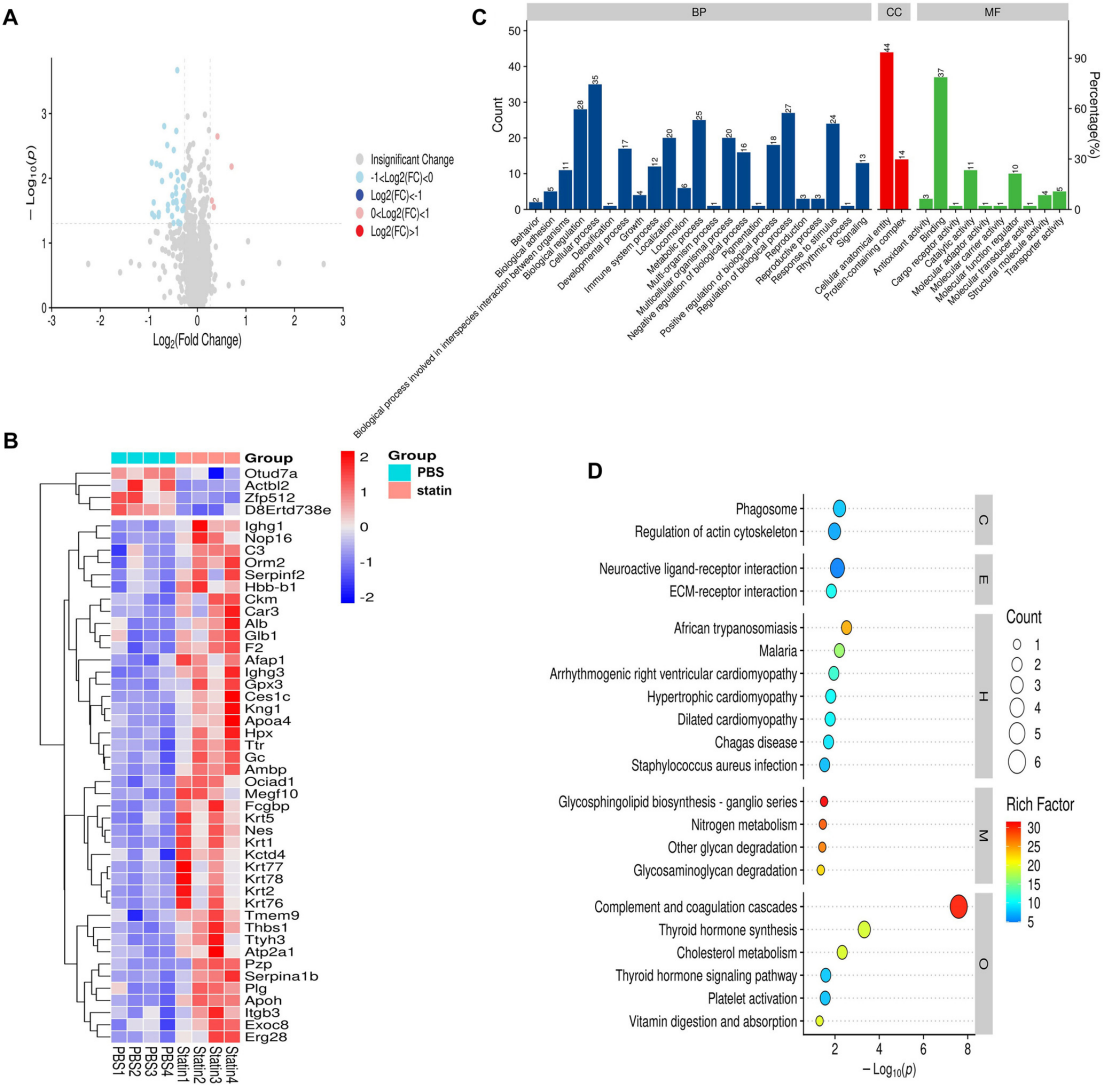
**DEPs in spinal cord tissues of mice between the SOD1^G93A^ + PBS and SOD1^G93A^ + simvastatin groups.** (A) Volcano plot showing DEPs in the SOD1^G93A^ + PBS and SOD1^G93A^ + simvastatin groups. Red dots indicate significantly upregulated proteins, while blue dots indicate significantly downregulated proteins in the SOD1^G93A^ + PBS group compared to the SOD1^G93A^ + simvastatin group. (B) Heatmap showing DEPs in the SOD1^G93A^ + PBS and SOD1^G93A^ + simvastatin groups. The red band represents upregulated proteins, while the blue band represents downregulated proteins. (C) GO analysis reveals that DEPs participated in key biological processes. (D) KEGG pathway enrichment analysis indicates that DEPs were mainly enriched in complement and coagulation cascades. SOD1: Superoxide dismutase 1; FC: Fold-change; GO: Gene Ontology; DEPs: Differentially expressed proteins; KEGG: Kyoto Encyclopedia of Genes and Genomes.

In the SOD1^G93A^ + simvastatin group, 47 DEPs were identified compared to the SOD1^G93A^ + PBS group, including 43 upregulated and 4 downregulated proteins (Figure 4A). GO function annotation showed that these DEPs were primarily involved in BP, including cellular processes, biological regulation, regulation of BPs, metabolic processes, response to stimulus, and localization. CC annotations included cellular anatomical entities and protein-containing complexes, while MF annotations included binding, catalytic activity, and MF regulation (Figure 4C).

KEGG pathway analysis identified significant involvement in pathways, such as African trypanosomiasis, malaria, glycosphingolipid biosynthesis-ganglio series, nitrogen metabolism, other glycan degradation, glycosaminoglycan degradation, complement and coagulation cascades, thyroid hormone synthesis, cholesterol metabolism, and others (Figure 4D).

### Metabolomic analysis

**Figure 5.**
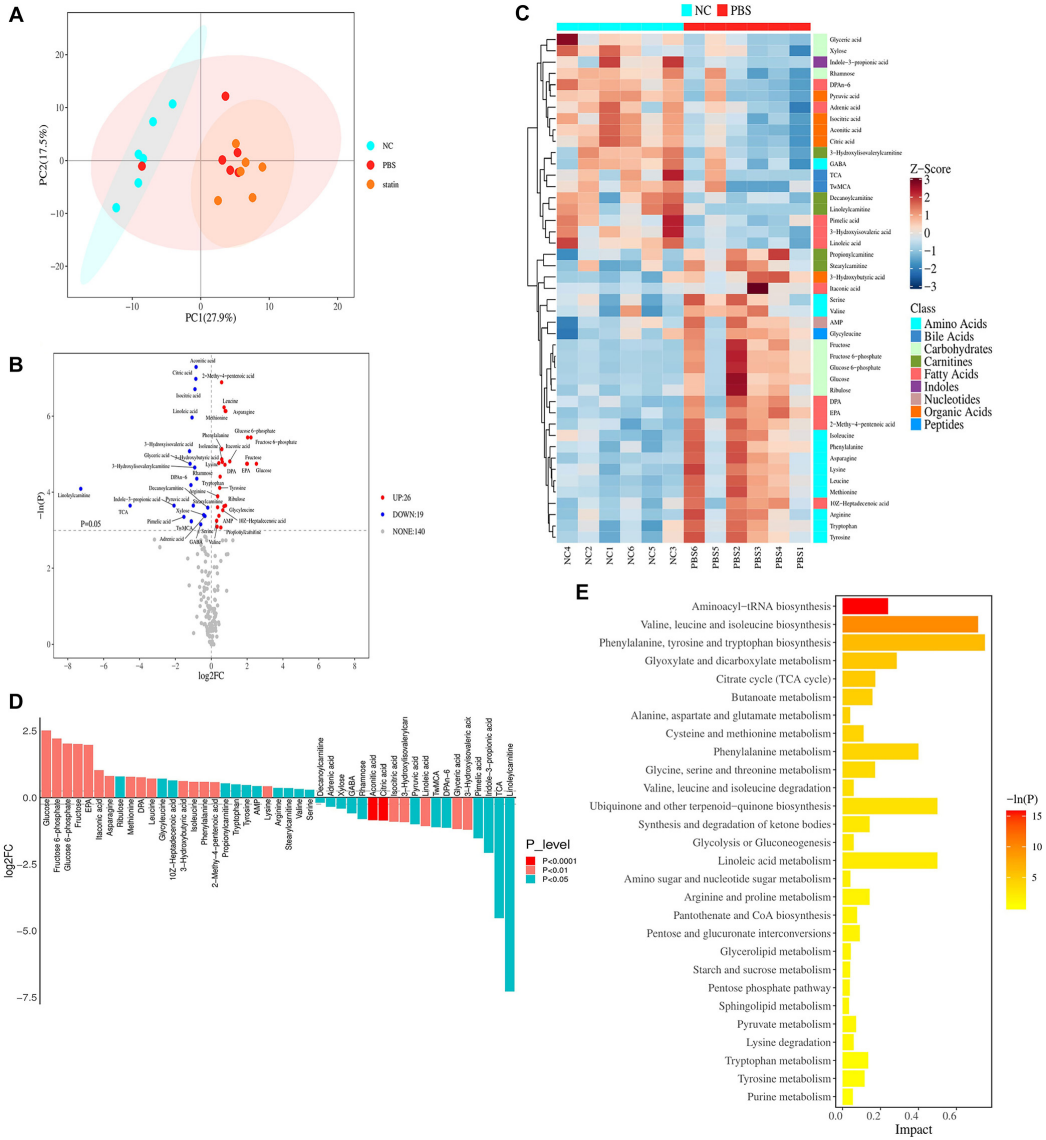
**Metabolic profile shift between the WT and SOD1^G93A^ + PBS groups.** (A) Principal component analysis presented the structural results among three groups. (B) Volcano plot of differential metabolites between the WT and SOD1^G93A^ + PBS groups. Red dots indicate metabolites significantly upregulated, while blue dots indicate metabolites significantly downregulated in the SOD1^G93A^ + PBS group compared with the WT group. (C) Heatmap showing differential metabolites between the WT and SOD1^G93A^ + PBS groups. The red band indicates upregulated metabolites, while the blue band indicates downregulated metabolites in the SOD1^G93A^ + PBS group compared with the WT group. (D) Histogram showing the GO enrichment results. (E) KEGG pathway enrichment revealed that the differential metabolites were primarily enriched in aminoacyl-tRNA biosynthesis. SOD1: Superoxide dismutase 1; WT: Wilt-type; FC: Fold-change; GO: Gene Ontology; KEGG: Kyoto Encyclopedia of Genes and Genomes.

PCA analysis revealed significant differences among the WT (NC) group, SOD1^G93A^ + PBS, and SOD1^G93A^ + simvastatin groups (Figure 5A). A total of 45 differential metabolites were identified, including 26 upregulated and 19 downregulated metabolites between the SOD1^G93A^ + PBS and WT groups (Figure 5B). Cluster analysis of potential biomarkers showed that distinct clusters were significantly enriched (Figure 5C). The upregulated differential metabolites included glucose, fructose 6-phosphate, glucose 6-phosphate, fructose, EPA, itaconic acid, asparagine, ribulose, methionine, DPA, etc., while the downregulated differential metabolites included aconitic acid, citric acid, isocitric acid, 3-hydroxylisovalerylcar1 acid, linoleic acid, glyceric acid, and 3-hydroxyisovaleric acid (Figure 5D). KEGG pathway enrichment analysis revealed that these metabolites were primarily enriched in aminoacyl-tRNA biosynthesis, valine, leucine, and isoleucine biosynthesis, among others (Figure 5E).

**Figure 6.**
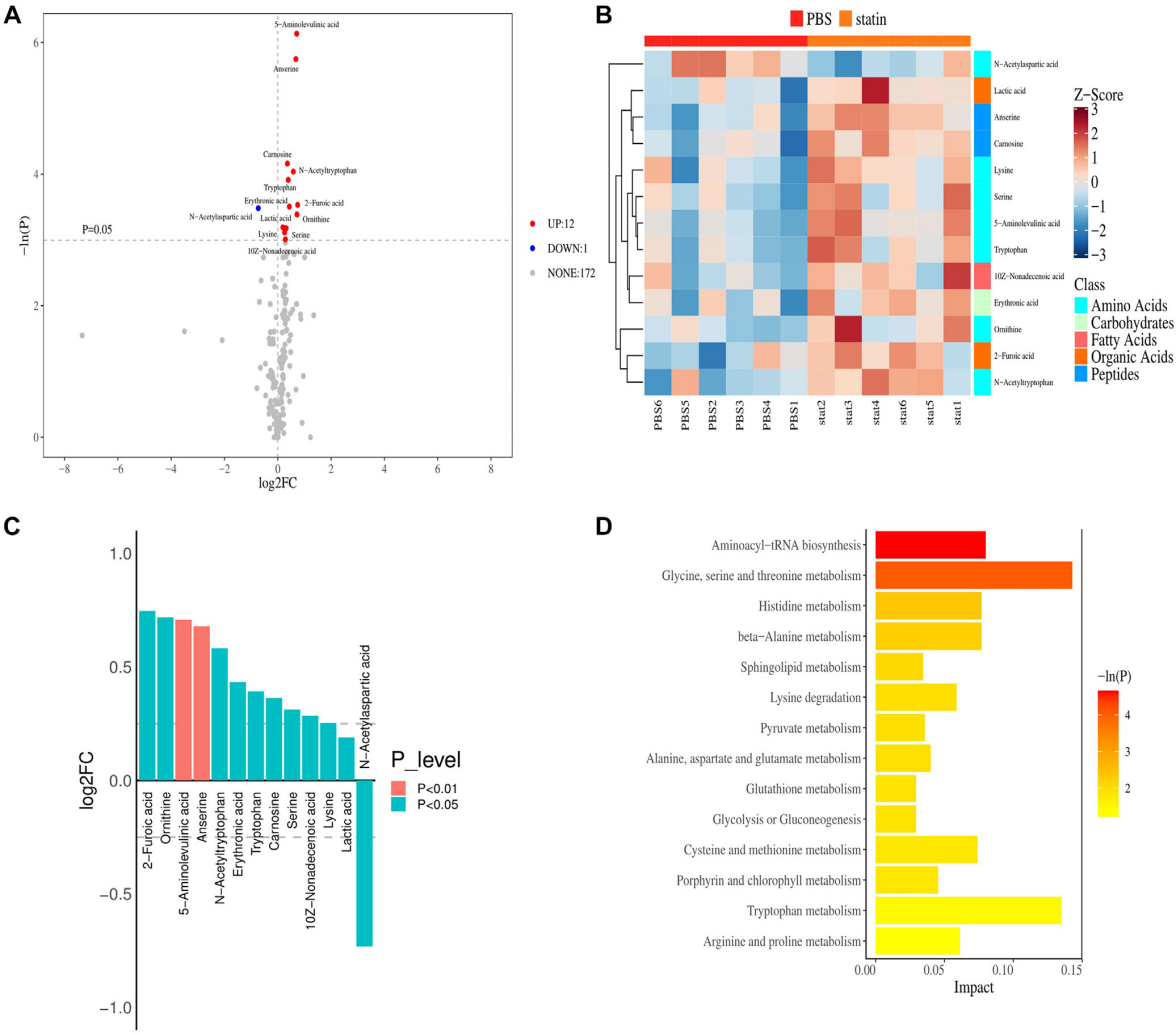
**Metabolic profile shift between the SOD1^G93A^ + PBS and SOD1^G93A^ + simvastatin groups.** (A) Volcano plot of differential metabolites between the SOD1^G93A^ + PBS and SOD1^G93A^ + simvastatin groups. Red dots indicate metabolites significantly upregulated, while blue dots indicate metabolites significantly downregulated in the SOD1^G93A^ + PBS group compared with the SOD1^G93A^ + simvastatin group. (B) Heatmap showing differential metabolites between the SOD1^G93A^ + PBS and SOD1^G93A^ + simvastatin groups. The red band indicates upregulated metabolites, while the blue band indicates downregulated metabolites in the SOD1^G93A^ + PBS group compared with the SOD1^G93A^ + simvastatin group. (C) Histogram showing the GO enrichment results. (D) KEGG pathway enrichment revealed that the differential metabolites were primarily enriched in aminoacyl-tRNA biosynthesis, glycine, serine, and threonine metabolism. SOD1: Superoxide dismutase 1; FC: Fold-change; GO: Gene Ontology; KEGG: Kyoto Encyclopedia of Genes and Genomes.

A total of 13 differential metabolites were identified, with 12 upregulated and 1 downregulated in the SOD1^G93A^ + simvastatin group compared to the SOD1^G93A^ + PBS group (Figure 6A). Cluster analysis revealed distinct enrichment of potential biomarkers between these groups (Figure 6B). The upregulated metabolites included 2-furoic acid, ornithine, 5-aminolevulinic acid, anserine, N-acetyltryptophan, erythronic acid, tryptophan, carnosine, serine, 10Z-nonadecenoic acid, lysine, and lactic acid, while the sole downregulated metabolite was N-acetylaspartic acid (NAA) (Figure 6C). GO and KEGG pathway enrichment analyses indicated that these metabolites were primarily enriched in aminoacyl-tRNA biosynthesis, glycine, serine, and threonine metabolism, and histidine metabolism, among others (Figure 6D).

### Expression levels of candidate genes

**Figure 7.**
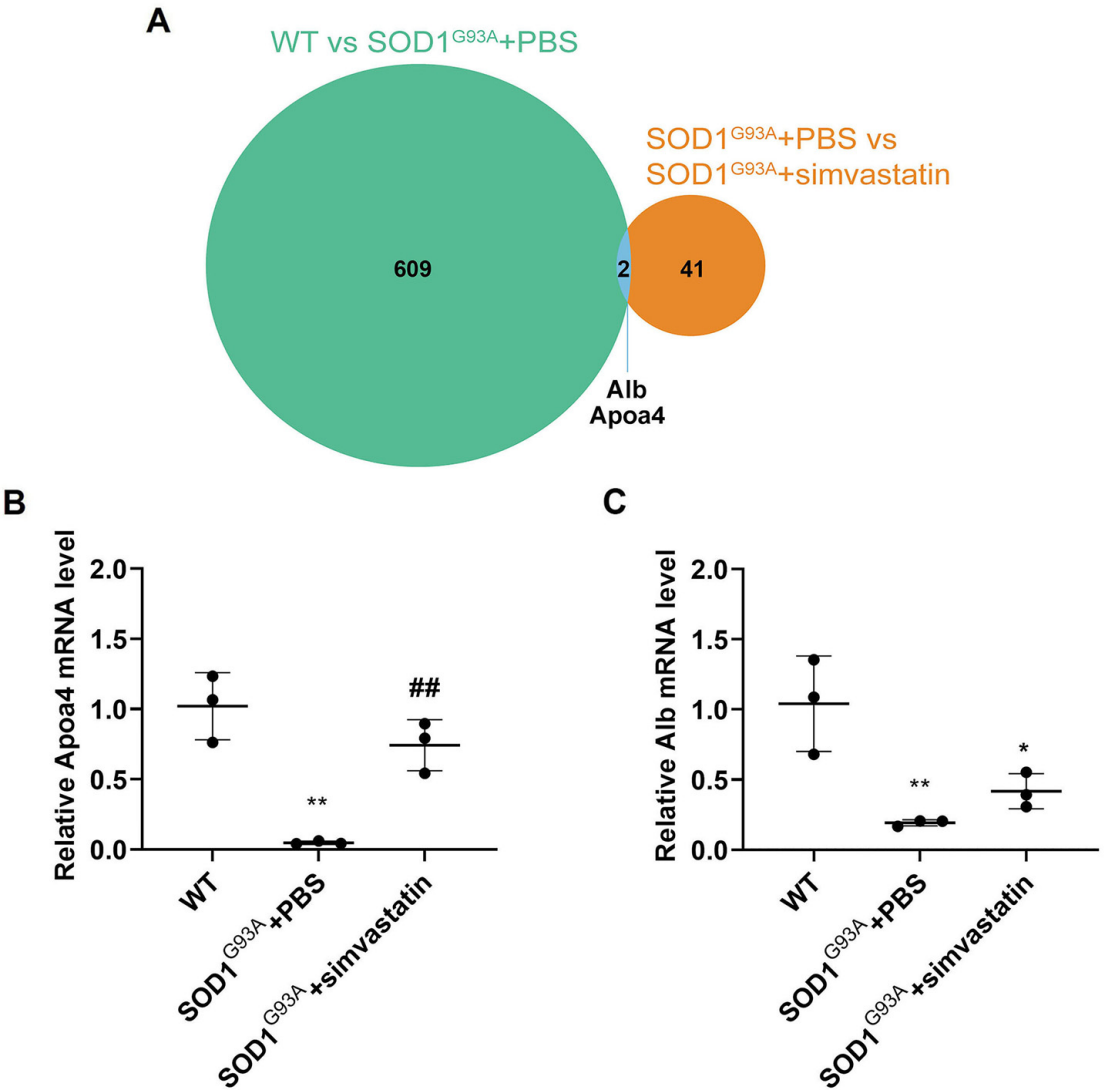
**Alb and Apoa4 expression levels in spinal cord tissues detected by qRT-PCR.** (A) Venn diagram showing the intersection of two genes obtained from the WT vs SOD1^G93A^ + PBS group and SOD1^G93A^ + PBS vs SOD1^G93A^ + simvastatin group; (B and C) The expression levels of Apoa4 (B) and Alb (C) decreased in the SOD1^G93A^ + PBS group compared with the WT group but increased in the SOD1^G93A^ + simvastatin group compared with the SOD1^G93A^ + PBS group. ^*^P < 0.05, ^**^P < 0.01, vs WT group; ^##^*P* < 0.01, vs SOD1^G93A^ + PBS group. SOD1: Superoxide dismutase 1; WT: Wilt-type; qRT-PCR: Quantitative real-time polymerase chain reaction.

Common targets were identified by comparing 611 downregulated proteins in the SOD1^G93A^ + PBS group and 43 upregulated proteins in the SOD1^G93A^ + simvastatin group using a Venn diagram (Figure 7A). Alb and Apoa4 were identified as common targets and selected as candidate genes. qRT-PCR results revealed that Apoa4 expression levels were significantly reduced in the SOD1^G93A^ + PBS group compared to the WT group (Figure 7B and 7C, *P* < 0.01). Apoa4 expression levels increased significantly in the SOD1^G93A^ + simvastatin group compared to the SOD1^G93A^ + PBS group (*P* < 0.05). However, Alb expression levels in the SOD1^G93A^ + simvastatin group showed no significant difference compared to the SOD1^G93A^ + PBS group (Figure 7C, *P* > 0.05).

### Downstream mechanisms-related genes and proteins were analyzed

**Figure 8.**
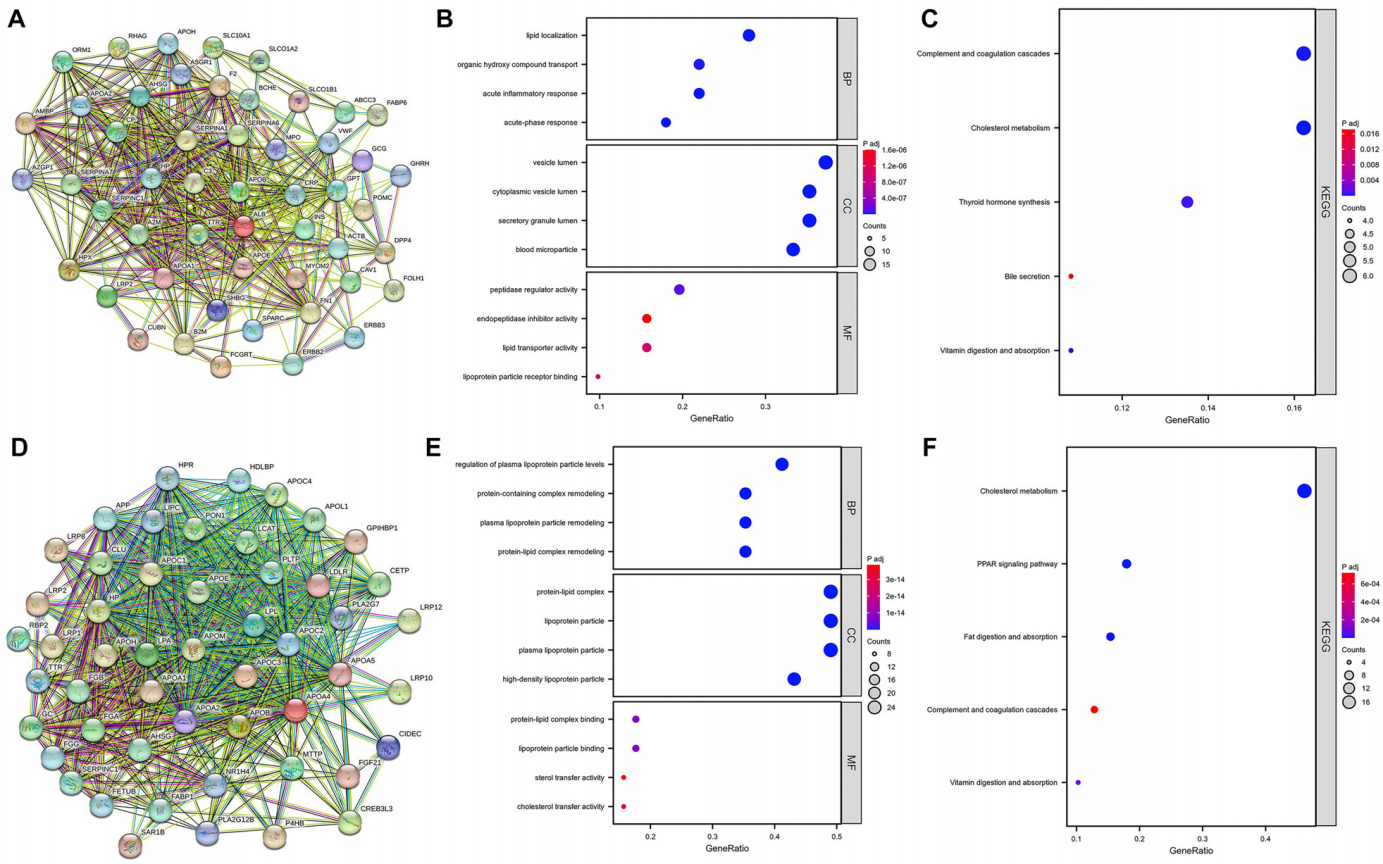
**PPI network and enrichment analysis.** (A) PPI network constructed for Alb; (B) GO analysis revealed that Alb participated in lipid localization, vesicle lumen, and peptidase regulator activity; (C) KEGG pathway annotation revealed that Alb was mainly enriched in complement and coagulation cascades and cholesterol metabolism; (D) PPI network constructed for Apoa4; (E) GO analysis revealed that Apoa4 participated in the regulation of plasma lipoprotein particle levels, protein–lipid complexes, and protein–lipid complex binding; (F) KEGG pathway annotation revealed that Apoa4 was mainly enriched in cholesterol metabolism, PPAR signaling pathway, fat digestion and absorption, and complement and coagulation cascades. GO: Gene Ontology; KEGG: Kyoto Encyclopedia of Genes and Genomes; PPI: Protein–protein interaction; PPAR: Peroxisome proliferator-activated receptor.

Alb and Apoa4 genes were further analyzed using the STRING database to collect protein–protein interaction (PPI) data and construct the PPI network. GO functional annotation and KEGG pathway analyses were performed (Figure 8A–8F). Results indicated that Alb was primarily enriched in pathways related to bile secretion, thyroid hormone synthesis, cholesterol metabolism, and complement and coagulation cascades. Apoa4 was enriched in pathways, including complement and coagulation cascades, fat digestion and absorption, the peroxisome proliferator-activated receptor (PPAR) signaling pathway, and cholesterol metabolism.

**Figure 9.**
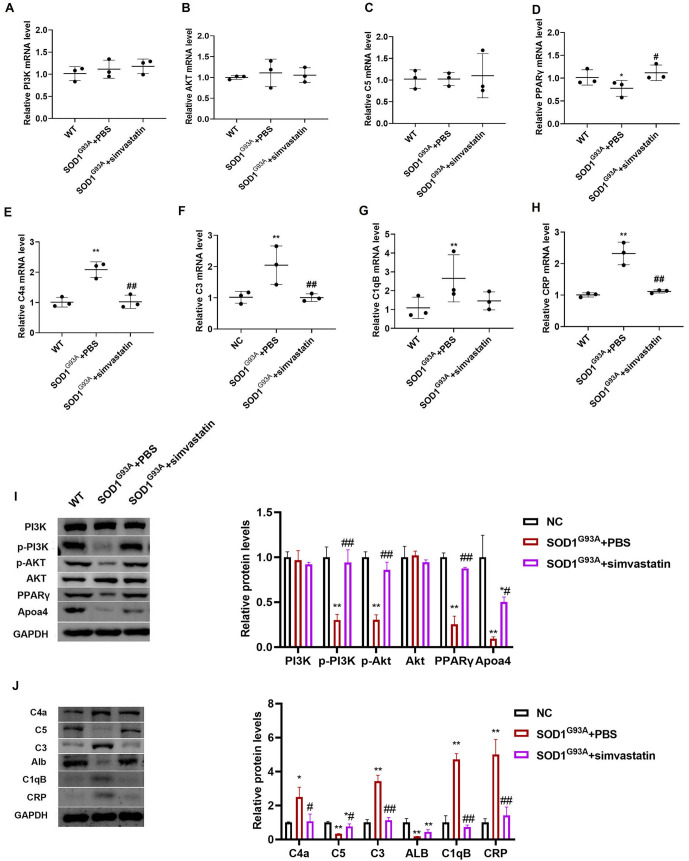
**Expression of mRNA and proteins.** qRT-PCR results presented the expression levels of PI3K (A), AKT (B), C5 (C), PPARγ (D), C4a (E), C3 (F), C1qB (G), and CRP (H) mRNA in different groups; the expression levels of PI3K, p-PI3K, p-AKT, AKT, PPARγ, Apoa4 (I), and C4a, C5, C3, Alb, C1qB, and CRP (J) in different groups were detected. The levels of p-PI3K, p-AKT, PPARγ, Apoa4, C5, and Alb decreased, while C4a, C3, C1qB, and CRP increased in the SOD1G93A + PBS group compared with the WT group; ^*^P < 0.05, ^**^P < 0.01, vs WT group; ^#^*P* < 0.05, ^##^*P* < 0.01, vs SOD1^G93A^ + PBS group. qRT-PCR: Quantitative real-time polymerase chain reaction; SOD1: Superoxide dismutase 1; WT: Wilt-type; PPAR: Peroxisome proliferator-activated receptor.

qRT-PCR results showed no significant differences in the expression levels of PI3K, AKT, and C5 mRNA among the three groups (Figure 9A–9C, *P* > 0.05). PPARγ expression was reduced in the SOD1^G93A^ + PBS group compared to the WT group but increased in the SOD1^G93A^ + simvastatin group, with no significant difference compared to the WT group (Figure 9D, *P* > 0.05). mRNA levels of C4a, C3, C1qB, and CRP were significantly elevated in the SOD1^G93A^ + PBS group compared to the WT group (Figure 9E–9H, *P* < 0.05). After simvastatin treatment, their levels significantly decreased.

Western blot analysis revealed reduced Apoa4 protein levels in the SOD1^G93A^ + PBS group compared to the WT group. However, simvastatin treatment significantly increased Apoa4 expression levels compared to the SOD1^G93A^ + PBS group. The levels of phosphorylated PI3K, phosphorylated AKT, and PPARγ were decreased in the SOD1^G93A^ + PBS group but increased following simvastatin treatment (Figure 9I, *P* < 0.05). Additionally, Alb protein levels were reduced in the SOD1^G93A^ + PBS group compared to the WT group and increased in the SOD1^G93A^ + simvastatin group. Protein levels of C4a, C3, C1qB, and CRP were elevated in the SOD1^G93A^ + PBS group but decreased following simvastatin treatment (Figure 9J, *P* < 0.05).

## Discussion

A previous study demonstrated that extended simvastatin treatment has the potential to enhance the regeneration of damaged muscle by activating the mammalian target of rapamycin pathway in ALS [8]. Bai et al. [4] proposed that simvastatin significantly exacerbates the impairment of late autophagic flux, leading to extensive motoneuron death in the spinal cord and hastening the progression of the disease in SOD1^G93A^ mice. Qi et al. [23] indicated that the impact of simvastatin on NSC34-hSOD1G93A cells may be attributed to the exacerbation of autophagic flux impairment through the inhibition of GGPP synthesis. At present, there is still controversy over whether simvastatin can be used for the treatment of ALS. Therefore, we attempted to analyze the changes in protein expression and metabolites in ALS mice treated with simvastatin through proteomics and metabolomics.

We found no significant differences in survival, climbing pole test scores, body weight, suspension test results, or neurological deficit scores between the SOD1^G93A^ + simvastatin group and the S SOD1^G93A^ + PBS group. The survival rate of the SOD1^G93A^ + simvastatin group was higher than that of the SOD1^G93A^ + PBS group from day 140 to day 160. These results differed from those of previous studies [4]. Furthermore, the decreasing trend in climbing pole test scores in the SOD1^G93A^ + simvastatin group was slower than that in the SOD1^G93A^ + PBS group from week 16 to week 18. Nevertheless, body weight, suspension test results, and neurological deficit scores showed no significant improvement or even a reduction after simvastatin treatment. Pathological findings, however, revealed the effectiveness of simvastatin treatment in SOD1^G93A^ mice. We then used spinal cord tissue to perform proteomics and metabolomics analyses. The results of the proteomics analysis showed that DEGs among the WT, SOD1^G93A^ + PBS, and SOD1^G93A^ + simvastatin groups were mainly enriched in coagulation cascades and cholesterol metabolism. Metabolomics analysis revealed that different metabolites belonged to five main groups: amino acids, carbohydrates, fatty acids, organic acids, and peptides. NAA was decreased in the SOD1^G93A^ + simvastatin group. NAA, a key component of the central nervous system, is synthesized in neurons from aspartate and acetyl-coenzyme A [24]. In ALS, the most prominent neurochemical changes are observed in the motor cortex and corticospinal tracts, indicating neuronal loss or dysfunction, specifically a decrease in NAA [25]. This result was contrary to the pathological findings. However, due to the small sample size, further confirmation is needed to explain this result. According to the proteomics results, the metabolomics data showed a distinct transition from acids and carbohydrates to lipids as the main source of energy. Apoa4 and Alb were increased after simvastatin treatment. Subsequent qRT-PCR and western blot results demonstrated that the administration of simvastatin led to an increase in both Apoa4 and Alb mRNA and protein expression levels.

The PPI network revealed potential connections between 50 genes and Apoa4 or Alb, respectively. KEGG pathway analysis indicated that Alb and Apoa4 are both enriched in cholesterol metabolism and complement and coagulation cascades. We chose the PI3K/AKT signaling pathway, complement and coagulation cascades, and PPAR signaling pathways for further validation. PI3K/AKT signaling is one of the important intracellular signaling pathways regulating basic cell functions and metabolism [26]. Several pathways are often dysregulated in neurodegeneration, including inflammation signaling, mitochondrial function, and lipid metabolism, which are regulated by the PPAR receptor [27]. Depending on the activation factors, the complement cascade is activated through three pathways: the classical pathway, the lectin pathway, and the alternative pathway [28]. Complement component C4 is pivotal in the activation cascades of complement pathways [29].

qRT-PCR results revealed that the expression levels of PPARγ decreased in the SOD1^G93A^ + PBS group but increased after simvastatin treatment. Additionally, the expression levels of C4a, C3, C1qB, and CRP were elevated in the SOD1^G93A^ + PBS group, whereas simvastatin treatment suppressed the expression levels of these proteins. Western blot results confirmed that the expression levels of p-PI3K, p-AKT, and C5 decreased in the SOD1^G93A^ + PBS group but were enhanced with simvastatin treatment. The protein expression levels of C4a, C3, C1qB, and CRP were consistent with the qRT-PCR results. CRP levels are known to be significantly increased in ALS patients [30]. Joardar et al. [31] provided evidence that while modulation of PPARγ activity does not improve lifespan, it represents a promising molecular target for alleviating locomotor dysfunction in Drosophila models of ALS specifically associated with TDP-43 and FUS, but not SOD1.

## Conclusion

Our study highlights the effectiveness of simvastatin treatment for ALS. Apoa4 and Alb were initially identified in relation to ALS. Notably, our results demonstrate that the effects of simvastatin treatment are associated with the PI3K/AKT signaling pathway, complement and coagulation cascades, and the PPAR signaling pathway. However, due to the limited number of mice used in the study, the subjective nature of behavioral testing may introduce a considerable degree of variability. Furthermore, fewer DEPs and metabolites were analyzed between the SOD1^G93A^ + PBS and SOD1^G93A^ + simvastatin groups. Future studies involving proteomics and metabolomics analyses in ALS patients are needed to confirm our findings.

## Supplemental data

**Table S1:** PCR primers and conditions

**Gene**	**Primer sequences (5′ to 3′)**
GAPDH	AGGTCGGTGTGAACGGATTTG
	GGGGTCGTTGATGGCAACA
Apoa4	CCAATGTGGTGTGGGATTACTT
	AGTGACATCCGTCTTCTGAAAC
Alb	CAAGAGTGAGATCGCCCATCG
	TTACTTCCTGCACTAATTTGGCA
PPARγ	GTAATCAGCAACCATTGGGTCA
	ACACCACGGTTTGGACTATGG
PI3K	GGCTACAGTAGTGGGCTTGG
	ATGAACGACGTAGCCATTGTG
AKT	TTGTAGCCAATAAAGGTGCCAT
	GTAATCAGCAACCATTGGGTCA
C3	CGGTGTGCTGAAGAGAACTG
	TTGATGACCTGCTGGATGGT
C5	TGTGCCAAAGAAATGCTGCT
	TGGATCCTTCCCAGTTGGAC
C4a	GGAAGGAAACAGCAAAGGCA
	GCGTCTGTGACCTTCACTTC
C1qB	GCAAGAGGAGGTTGTTCACC
	CAGTGAAGATGCTGTTGGCA
CRP	CGCAGCTTCAGTGTCTTCTC
	AGATGTGTGTTGGAGCCTCA

PPAR: Peroxisome proliferator-activated receptor.

## Data Availability

The data that support the findings of this study are available from the corresponding author upon reasonable request.
